# Deformable image registration for adaptive radiotherapy with guaranteed local rigidity constraints

**DOI:** 10.1186/s13014-016-0697-4

**Published:** 2016-09-20

**Authors:** Lars König, Alexander Derksen, Nils Papenberg, Benjamin Haas

**Affiliations:** 1 Fraunhofer MEVIS, Maria-Goeppert-Str. 3, Lübeck, 23562 Germany; 2 Varian Medical Systems, Imaging Laboratory GmbH, Täfernstr. 7, Baden, 5405 Switzerland

**Keywords:** Deformable image registration, Local rigidity, Adaptive radiotherapy, CT, CBCT, Prostate cancer

## Abstract

**Background:**

Deformable image registration (DIR) is a key component in many radiotherapy applications. However, often resulting deformations are not satisfying, since varying deformation properties of different anatomical regions are not considered. To improve the plausibility of DIR in adaptive radiotherapy in the male pelvic area, this work integrates a local rigidity deformation model into a DIR algorithm.

**Methods:**

A DIR framework is extended by constraints, enforcing locally rigid deformation behavior for arbitrary delineated structures. The approach restricts those structures to rigid deformations, while surrounding tissue is still allowed to deform elastically. The algorithm is tested on ten CT/CBCT male pelvis datasets with active rigidity constraints on bones and prostate and compared to the Varian SmartAdapt deformable registration (VSA) on delineations of bladder, prostate and bones.

**Results:**

The approach with no rigid structures (REG0) obtains an average dice similarity coefficient (DSC) of 0.87 ± 0.06 and a Hausdorff-Distance (HD) of 8.74 ± 5.95 mm. The new approach with rigid bones (REG1) yields a DSC of 0.87 ± 0.07, HD 8.91 ± 5.89 mm. Rigid deformation of bones and prostate (REG2) obtains 0.87 ± 0.06, HD 8.73 ± 6.01 mm, while VSA yields a DSC of 0.86 ± 0.07, HD 10.22 ± 6.62 mm. No deformation grid foldings are observed for REG0 and REG1 in 7 of 10 cases; for REG2 in 8 of 10 cases, with no grid foldings in prostate, an average of 0.08 % in bladder (REG2: no foldings) and 0.01 % inside the body contour. VSA exhibits grid foldings in each case, with an average percentage of 1.81 % for prostate, 1.74 % for bladder and 0.12 % for the body contour. While REG1 and REG2 keep bones rigid, elastic bone deformations are observed with REG0 and VSA. An average runtime of 26.2 s was achieved with REG1; 31.1 s with REG2, compared to 10.5 s with REG0 and 10.7 s with VMS.

**Conclusions:**

With accuracy in the range of VSA, the new approach with constraints delivers physically more plausible deformations in the pelvic area with guaranteed rigidity of arbitrary structures. Although the algorithm uses an advanced deformation model, clinically feasible runtimes are achieved.

**Electronic supplementary material:**

The online version of this article (doi:10.1186/s13014-016-0697-4) contains supplementary material, which is available to authorized users.

## Background

In adaptive radiotherapy, deformable image registration is a key component in many applications, including e.g. contour propagation and dose accumulation [1].

A common assumption of clinically available registration frameworks are homogeneous deformation properties of tissue in the image domain [2]. However, especially in the pelvic region, there are several risk structures directly adjacent that possess widely varying deformation properties [3, 4]. While rectum and bladder deform highly elastic, individual bones deform nearly rigid and comparably stiff movement of the prostate may be observed [5]. Here, deformable image registration (DIR) algorithms can be beneficial compared to rigid alignment [6], but still do not yield acceptable results frequently [7]. This motivates incorporation of improved deformation models [8, 9]. Several different approaches have been pursued, including biomechanical models [10, 11], contour guided registration [12] and shape based regularization [13]. In pelvis images, however, especially bone structures are often deformed in non-plausible ways [7]. Approaches with locally rigid deformation behavior have been presented for a general medical imaging context [14–17], and also specifically for the radiotherapy context [18, 19].

In this work, we present a clinically applicable extension of a variational DIR framework [20], enabling rigid deformation of arbitrary structures, embedded in a deformable registration. With an image distance term based on normalized gradient fields [21], which has been proven successful in a wide range of applications, including radiotherapy [22–25], the proposed method is well suited for multi-modal CT to cone-beam CT (CBCT) registrations. In contrast to previously presented approaches, this algorithm is based on a variational model and implements local rigidity as a hard constraint, in which rigidity is guaranteed.

The contribution of this work is an extension and adaption of the theoretical framework described in [15] to clinically relevant three-dimensional data, embedded in a powerful registration scheme, allowing for clinically feasible runtimes. The presented approach is evaluated on ten pelvis CT/CBCT datasets in comparison with the demons-based DIR algorithm of Varian SmartAdapt (VSA) [26].

## Methods

### Image registration algorithm

As basis for the registration algorithm, a highly parallel registration framework with low memory consumption has been used [23, 25]. In the following, a general description of the registration algorithm is given, a more detailed mathematical description of the presented framework is part of the Additional file 1 of this article.

The registration approach determines an optimal transformation *y* between two images through the minimization of a joint objective function

1 $$\begin{array}{*{20}l} J(y)= D_{\text{NGF}}(I_{\text{CT}}, I_{\text{CBCT}}(y)) + \alpha S_{\text{Curv}}(y) \end{array} $$

by using a standard limited-memory Broyden-Fletcher-Goldfarb-Shanno (L-BFGS) optimization scheme [27]. Here, the first term *D*_NGF_(*I*_CT_,*I*_CBCT_(*y*)) represents the normalized gradient fields distance measure, depending on the CT image *I*_CT_ and the deformed CBCT image *I*_CBCT_(*y*). This distance measure is based on the assumption that in two given images, regardless of modality, object edges and boundaries are always represented by intensity changes, i.e. image gradients and tries to align those image edges. It features two edge filtering parameters *τ*,*ϱ* that are used to determine which edges are interpreted as noise for *I*_CT_ and *I*_CBCT_, respectively.

The second term *S*_Curv_ is a curvature regularizer [28], which is based on second order derivatives and thus favors smooth deformation fields. The regularizer is weighted by parameter *α*, which can be used to balance between smooth deformations and image similarity. Considering that rigid structures will be embedded in the deformation field, second order curvature regularization has been chosen over more common and less smooth first order regularization approaches [20].

The algorithm is embedded in a multi-level scheme, where the problem is solved on coarser to fine resolutions, each using the result of the previous level as a starting guess.

This framework, however, does not incorporate any additional knowledge about special anatomical deformation properties. Especially in structures such as bones, this can lead to physically implausible deformations, which impact the credibility and thus the clinical use of DIR.

### Local rigidity

Therefore, as proposed in [15], this framework is extended to feature locally rigid deforming structures, embedded in the deformable transformation. Delineations of the structures that should be kept rigid are assumed on *I*_CT_, no delineations are required on *I*_CBCT_. By substituting each point of the deformation *y* that is inside a rigid area by using a rigid transformation *Q*(*θ*)+*b*, with a rotational matrix and angles *Q*(*θ*) and translation vector *b*, the transformation can be reformulated as *y*=(*y*_0_,*y*_1_,…,*y*_*M*_)=(*y*_0_,*Q*(*θ*_1_)*x*+*b*_1_,…,*Q*(*θ*_*M*_)*x*+*b*_*M*_). Here, *y*_0_ describes the deformation of all points outside the rigid areas and thus should deform non-rigidly, while *y*_1_,…,*y*_*M*_ describe the *M* independent rigid deformations of points inside the rigid areas. Substituting this in the objective function (1), the optimization problem becomes unconstrained again, now optimizing over parameters *y*_0_, containing all unconstrained points, and rotational angles plus translation vectors *θ*_*k*_,*b*_*k*_,*k*=1,…,*M*. This can then be solved using the same optimization scheme as before.

In this approach, rigid deformations of the defined regions are *guaranteed*. Furthermore, the rigidity integrates directly into the established unconstrained optimization, there are no additional model parameters introduced that must be chosen and there are no specialized optimization strategies required such that fast standard techniques can be used.

### Dataset

To assess the developed algorithm, evaluation has been performed on a set of ten prostate cancer datasets from clinical routine including six cases with a rectal balloon (Case 0,2,3,4,5,6). These datasets were chosen retrospectively out of already existing cases from the hospital database and fully anonymized before being made available to the authors. All patients had given their written informed consent before radiotherapy. The research having been carried out here is in compliance with the declaration of Helsinki. These datasets consist of a single planning CT image and one CBCT acquired during the course of the fractionated treatment. The CT planning images have been acquired using a Philips Brilliance Big Bore CT scanner. All CT images were acquired/reconstructed with 512 columns, 512 rows with an average in-plane resolution of 0.98 ×0.98 mm ^2^ and an average number of 238 slices with a slice thickness/spacing of 2 mm for Case 0,1,4,6,7,8,9 and a slice thickness/spacing of 1 mm for Case 2,3,5 (120 kVp, 271-325 mA, average 297 mA, helical mode, convolution kernel B, Philips Healthcare, Best, The Netherlands).

The CBCT images were acquired/reconstructed with a Varian TrueBeam On-Board Imager with 512 columns, 512 rows with an in-plane resolution of 0.91 ×0.91 mm ^2^ and 81 slices with a slice thickness/spacing of 1.99 mm (125 kV, 80 mA, convolution kernel Ram-Lak).

The CT images have been manually delineated with structures of at least femoral heads, pelvic bones, prostate (CTV) and bladder for daily clinical use. For evaluation purposes, a single CBCT image has been selected randomly for each case. On this CBCT image, additional manual delineations of the aforementioned structures have been performed by a radiation oncologist, which serve as a gold standard.

### Evaluation

First, CT and CBCT images were registered using an automatic rigid pre-alignment (RIG). Subsequently, based on the rigid alignment, the images were registered using two different DIR algorithms. First, the standard DIR implementation of Varian SmartAdapt (VSA) is used (no tunable parameters). Second, the presented algorithm is evaluated using three different sets of structures that are kept rigid. For the first set of rigid structures, an empty delineation was used, i.e. no constraints are active (REG0). For the second set, all bones are defined as rigid structures (REG1) and for the third set, additionally to the bones, the prostate is also defined as rigid structure during registration (REG2).

To determine suitable parameters *α*,*τ* and *ϱ*, several parameter combinations were examined. We tested values of [1,5,10,20,50] for *α* and values of [1,5,10] for *τ* and *ϱ* in all possible combinations and chose the parameters with best DSC values. The finest deformation level that was calculated was discretized with an average number of 105 ×65×41 grid points leading to average grid spacings of 3.65×3.64×4.01 mm ^3^. Using the resulting deformations, delineations of prostate (CTV), bladder and right femoral head were propagated to the CBCT for comparison with the manually delineated gold standard.

Comparison of the propagated structures was performed by using the Dice similarity coefficient (DSC), which measures the spatial overlap of contoured structures [29] and Hausdorff distance (HD) [30], which is defined as the maximum of all closest distances from each point on one surface to all points on the other surface.

Besides accurately propagated delineations, displacement regularity is a necessary metric to determine physical plausibility of the computed deformations [31]. Especially “twists” or “foldings” in the deformation grid correspond to physically incorrect deformations, which directly impact the quality of the registration result [31].

To examine such foldings in the computed results, the Jacobian of the deformation det(∇*y*) was utilized. For det(∇*y*)=1 the transformation is volume preserving, while larger values indicate increase in volume and lower values indicate shrinkage. Computation of the Jacobian took place as described in [32], which is essential since only proper discretization ensures that all grid foldings (i.e. det(∇*y*)<0) can be detected [33].

The number of grid foldings was evaluated inside delineations of prostate (CTV), bladder and the body outline including the aforementioned structures. Furthermore, average Jacobian values were computed for structures where local rigidity was applied, i.e. prostate and right femoral head. All calculations were performed on a 12-core Intel Xeon E5-2620 workstation.

### Statistics

To test whether rectal balloons had an influence on the results, a two-sided two-sample t-test, comparing DSC values for cases with rectal balloon versus cases without rectal balloon separately for each DIR algorithm and parameterization has been performed. Additionally, tests were performed comparing DSC results of RIG with the DIR approaches, as well as comparing VSA with REG0, REG1 and REG2. To analyze statistical significance, a two-sided paired-sample t-test was chosen, corresponding to the assumption that the differences in the DSC values of different algorithms are normally distributed. Bonferroni correction was applied for definition of significance (*p*< 0.0045).

## Results

### Parameterization

Resulting from the parameter search described in the previous section, for REG0, REG1 and REG2 the parameters *α*=10, *τ*,*ϱ*=5 were used for all evaluations.

### Segmentation overlap

The resulting DSC and HD values are presented in Tables 1 and 2, respectively. Averaged over all structures, for the VSA algorithm a DSC of 0.86 ± 0.07 and a HD of 10.22 ± 6.62 mm was obtained, while REG0 resulted in a DSC of 0.87 ± 0.06 and a HD of 8.74 ± 5.95 mm.

**Table 1:** Comparison of the new method to the Varian SmartAdapt deformable registration in terms of DSC

Prostate						Bladder					Right femoral head				
ID	Rigid	VSA	REG0	REG1	REG2	Rigid	VSA	REG0	REG1	REG2	Rigid	VSA	REG0	REG1	REG2
0	0.79	0.81	0.85	0.84	0.82	0.84	0.91	0.90	0.91	0.91	0.94	0.94	0.96	0.96	0.96
1	0.80	0.89	0.85	0.85	0.85	0.83	0.91	0.86	0.86	0.86	0.89	0.94	0.94	0.95	0.95
2	0.53	0.71	0.75	0.71	0.75	0.66	0.87	0.86	0.85	0.85	0.89	0.91	0.91	0.91	0.91
3	0.74	0.70	0.80	0.80	0.79	0.76	0.85	0.89	0.89	0.89	0.89	0.92	0.93	0.93	0.93
4	0.70	0.73	0.81	0.81	0.81	0.74	0.82	0.80	0.80	0.80	0.92	0.94	0.94	0.94	0.94
5	0.82	0.82	0.85	0.85	0.87	0.82	0.87	0.92	0.92	0.92	0.89	0.93	0.94	0.94	0.94
6	0.84	0.81	0.84	0.84	0.84	0.83	0.88	0.84	0.84	0.84	0.90	0.89	0.90	0.90	0.90
7	0.71	0.82	0.85	0.85	0.85	0.49	0.75	0.78	0.78	0.78	0.91	0.95	0.97	0.98	0.98
8	0.70	0.86	0.77	0.77	0.82	0.63	0.81	0.83	0.83	0.80	0.93	0.95	0.95	0.95	0.95
9	0.76	0.81	0.79	0.80	0.79	0.80	0.89	0.89	0.89	0.89	0.90	0.94	0.95	0.95	0.95
Avg	0.74	0.80	0.82	0.81	0.82	0.74	0.86	0.86	0.86	0.85	0.91	0.93	0.94	0.94	0.94
Std	0.09	0.06	0.04	0.05	0.04	0.11	0.05	0.04	0.05	0.05	0.02	0.02	0.02	0.02	0.02

For ten cases, contours were propagated from CT to CBCT using the computed deformation. DSC values in comparison with expert provided delineations are given for each structure and each case. *Abbreviations*: “*Rigid*”: rigid pre-alignment; “*VSA*”: Varian SmartAdapt deformable registration; “*REG0*”: new approach without local rigidity; “*REG1*”: new approach with rigid bones; “*REG2*”: new approach with rigid bones and prostate; “*Avg*”/“*Std*”: average/standard deviation over all cases for each method and structure

**Table 2:** Comparison of the new method to the Varian SmartAdapt deformable registration in terms of HD

Prostate						Bladder					Right femoral head				
ID	Rigid	VSA	REG0	REG1	REG2	Rigid	VSA	REG0	REG1	REG2	Rigid	VSA	REG0	REG1	REG2
0	7.20	5.45	5.80	6.04	5.51	11.08	8.52	6.04	6.04	8.16	5.66	3.98	3.98	3.98	3.98
1	7.87	6.04	6.49	6.49	6.49	11.19	8.00	8.76	8.33	8.52	5.45	4.18	4.54	10.44	4.54
2	12.77	10.93	13.98	13.98	13.98	18.21	13.12	9.95	11.78	9.79	8.33	6.04	7.11	7.50	7.50
3	7.22	10.02	7.50	6.99	7.50	13.13	14.78	11.94	9.40	9.40	4.52	5.28	3.98	3.98	4.14
4	11.09	10.60	8.41	8.41	8.36	26.36	23.49	24.46	24.46	24.78	4.96	4.37	4.46	4.37	4.37
5	6.66	18.87	8.08	8.08	8.84	9.44	19.31	6.04	6.04	8.08	6.41	4.18	4.37	4.37	4.37
6	6.56	9.27	8.75	8.61	6.99	11.94	11.27	12.12	12.12	13.40	4.96	6.42	5.45	5.97	5.45
7	11.27	13.53	9.34	9.65	9.16	34.43	32.48	31.31	31.20	31.57	4.96	3.83	3.63	3.27	2.87
8	10.32	6.50	7.50	7.50	6.24	14.40	15.92	10.03	10.03	10.15	4.96	5.54	4.37	4.47	4.37
9	12.15	10.81	11.09	11.09	10.75	9.78	9.94	8.74	8.74	8.74	4.73	4.08	3.98	3.98	3.98
Avg	9.31	10.20	8.69	8.68	8.38	16.00	15.68	12.94	12.81	13.26	5.49	4.79	4.59	5.23	4.56
Std	2.44	3.97	2.37	2.39	2.51	8.22	7.65	8.30	8.32	8.17	1.13	0.94	1.01	2.20	1.21

See Table 1 for further description, HD values in mm

For the approaches using local rigidity, REG1 resulted in a DSC of 0.87 ± 0.07 and HD of 8.91 ± 5.89 mm and REG2 obtained a DSC of 0.87 ± 0.06 and a HD of 8.73 ± 6.02 mm.

Comparing the cases with no rectal balloon versus cases with rectal balloon did not show a statistical significant difference for VSA (*p*=0.36, 95 % CI -0.082–0.031), REG0 (*p*=0.94, 95 % CI -0.047–0.051), REG1 (*p*=0.98, 95 % CI -0.052–0.051) or REG2 (*p*=0.95, 95 % CI -0.050–0.047).

Using the initial rigid registration (RIG), an average DSC of 0.79 ± 0.11 and a HD of 12.65 ± 6.83 mm was achieved. Compared with these results, all approaches showed a statistical significant increase in DSC overlap (*p*<10^−5^, *p*<10^−5^, *p*<10^−5^, *p*<10^−4^ with 95 % CIs 0.050–0.102, 0.050–0.100, 0.051–0.102 and 0.040–0.092 for REG0, REG1, REG2 and VSA, respectively). Comparing the results of the DIR algorithms, statistical significant difference in comparison with the results of VSA could not be shown for REG0 (*p*=0.18, 95 % CI -0.005–0.024), REG1 (*p*=0.21, 95 % CI -0.005–0.023) or REG2 (*p*=0.10, 95 % CI −0.002–0.023).

### Displacement regularity

The percentage of grid foldings for prostate, bladder and the body outline for VSA, REG0, REG1 and REG2 is shown in Table 3. As can be seen, the new algorithm avoids grid foldings in eight/seven out of ten cases, while the VSA algorithm exhibits grid foldings in all ten cases. Furthermore, REG2 only contains foldings outside of bladder and prostate and only for two cases, while VSA also exhibits foldings in bladder or prostate in five cases.

**Table 3:** Fraction of deformation grid foldings per organ and case in percent

ID	Prostate				Bladder				Body			
	VSA	REG0	REG1	REG2	VSA	REG0	REG1	REG2	VSA	REG0	REG1	REG2
0	0.00	0.00	0.00	0.00	0.00	0.00	0.00	0.00	0.19	0.01	0.02	0.04
1	0.00	0.00	0.00	0.00	0.00	0.00	0.00	0.00	0.01	0.00	0.00	0.00
2	0.00	0.00	0.00	0.00	1.61	0.00	0.00	0.00	0.13	0.00	0.00	0.00
3	5.46	0.00	0.00	0.00	0.04	0.00	0.00	0.00	0.18	0.00	0.00	0.00
4	11.97	0.00	0.00	0.00	4.21	0.00	0.00	0.00	0.45	0.00	0.00	0.00
5	0.00	0.00	0.00	0.00	11.43	0.00	0.00	0.00	0.15	0.00	0.00	0.00
6	0.68	0.00	0.00	0.00	0.00	0.00	0.00	0.00	0.10	0.00	0.00	0.00
7	0.00	0.00	0.00	0.00	0.06	0.00	0.00	0.00	0.05	0.04	0.03	0.04
8	0.00	0.00	0.00	0.00	0.00	0.82	0.82	0.00	0.07	^a^0.00	^a^0.00	0.00
9	0.00	0.00	0.00	0.00	0.00	0.00	0.00	0.00	0.03	0.00	0.00	0.00
Avg	1.81	0.00	0.00	0.00	1.74	0.08	0.08	0.00	0.14	0.01	0.01	0.01
Std	3.75	0.00	0.00	0.00	3.48	0.25	0.25	0.00	0.12	0.01	0.01	0.02

Region specific fraction of volume percent involved in grid foldings of the computed deformation for different approaches and ten cases. Marked (^a^) values contain a small amount of grid foldings which rounds to zero. *Abbreviations*: “*VSA*”: Varian SmartAdapt deformable registration; “*REG0*”: new approach without local rigidity; “*REG1*”: new approach with rigid bones; “*REG2*”: new approach with rigid bones and prostate; “*Avg*”/“*Std*”: average/standard deviation over all cases for each method and structure

Additionally, average values of the Jacobian, determined on the computed deformations of VSA, REG0, REG1 and REG2 are evaluated for prostate and right femoral head in Table 4. As can be seen, the new algorithm with rigidity achieves fully rigid structures, while the VSA algorithm and REG0 exhibit elastic deformation of bones.

**Table 4:** Average Jacobian values of the computed deformations, evaluated inside the prostate and right femoral head

ID	Prostate				Right femoral head			
	VSA	REG0	REG1	REG2	VSA	REG0	REG1	REG2
0	0.92	0.94	0.93	1.00	0.90	0.97	1.00	1.00
1	0.94	0.94	0.93	1.00	0.95	0.98	1.00	1.00
2	1.02	0.74	0.74	1.00	1.04	0.96	1.00	1.00
3	1.02	1.02	1.04	1.00	0.95	0.92	1.00	1.00
4	0.76	1.05	1.05	1.00	0.99	0.98	1.00	1.00
5	0.89	0.92	0.89	1.00	0.95	0.96	1.00	1.00
6	0.89	1.11	1.13	1.00	0.90	0.99	1.00	1.00
7	0.84	1.00	0.99	1.00	1.02	0.95	1.00	1.00
8	0.85	0.67	0.68	1.00	1.01	0.95	1.00	1.00
9	0.99	0.97	1.06	1.00	1.04	0.93	1.00	1.00
Avg	0.91	0.94	0.94	1.00	0.98	0.96	1.00	1.00
Std	0.08	0.13	0.14	0.00	0.05	0.02	0.00	0.00

Region specific average Jacobian values of the computed deformation for different approaches and ten cases. “*VSA*”: Varian SmartAdapt deformable registration; “*REG0*”: new approach without local rigidity; “*REG1*”: new approach with rigid bones; “*REG2*”: new approach with rigid bones and prostate; “*Avg*”/“*Std*”: average/standard deviation over all cases for each method and structure

**Fig. 1:**
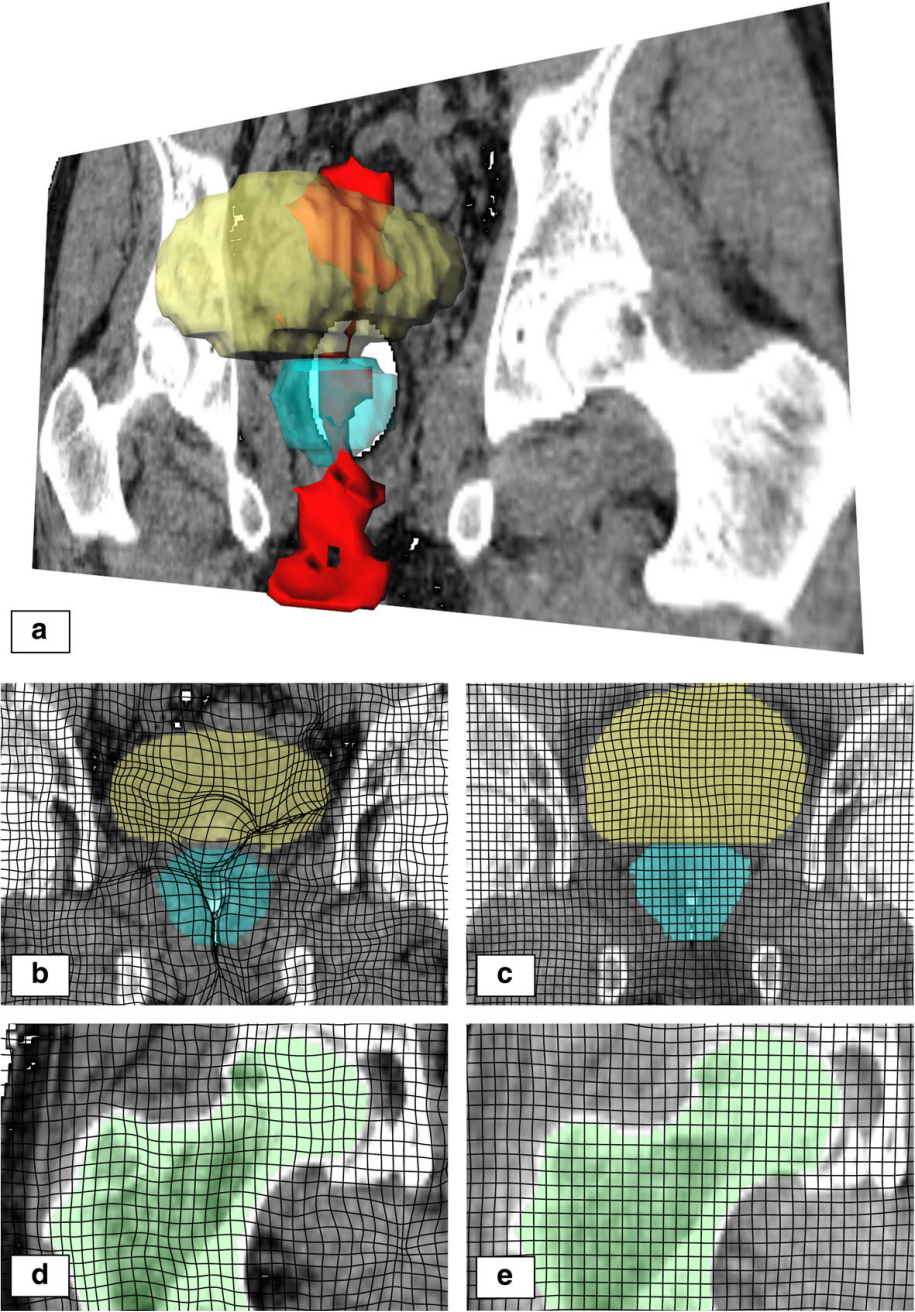
Visualization of displacement regularity in Case 4. **a** shows a 3D-visualization of the CT image (single coronal slice) with bladder (*yellow*), prostate (*blue*) and detected grid foldings generated by the VSA algorithm (*red*). **b**–**e** show close-up coronal projections of the deformation field of the VSA algorithm (*left*) and the REG2 algorithm (*right*) on prostate/bladder **b**/**c** and right femoral head **d**/**e**

Details of the deformation of Case 4 are visualized in Fig. 1. Here, in Fig. 1a a 3D-visualization of the CT image of Case 4 is shown. In addition to a single coronal slice, volumes of bladder, prostate and locations of grid foldings, generated by the VSA algorithm, are visualized. It can easily be seen that grid foldings are not limited to peripheral areas but are also occurring inside and at the boundary of prostate and bladder. In Fig. 1b and c a coronal slice of the deformation field is shown, containing masks of the bladder and prostate. Here, the VSA algorithm generates grid foldings, while the new algorithm computes a smooth deformation grid.

In addition to grid foldings, physical plausibility is also important from a different point of view. Computed deformations are expected to deform different structures according to their physical properties. Especially rigid structures like bones should not deform elastically. In Fig. 1d and e coronal slices of registration results in the area of the right femoral head are shown. Here, it can be seen that the VSA algorithm does not distinguish between bones and surrounding tissue, while the new algorithm keeps the femoral head rigid.

### Runtime

Using both fully deformable algorithms, comparable average runtimes of 10.7 s for VMS and 10.5 s for REG0 were achieved. The computations required for the local rigidity framework resulted in average runtimes of 26.2 s for REG1 and 31.1 s for REG2.

## Discussion

The proposed registration concept of integration of rigid structures into DIR in radiotherapy has already been highlighted by a few publications in recent years. In [19], an approach has been introduced that keeps bones rigid. More recently Kim et al. published a DIR framework that aims at establishing a benchmark CT/CBCT registration and incorporates a local rigidity constraint [18]. The above mentioned approaches have in common that they are based on a B-spline framework as introduced by Rueckert et al. [34] and implement local rigidity as a soft penalty, such that the condition might not be fulfilled in the resulting deformation. Our proposed approach differs in that sense, that it is based on a variational model as described in [20] and incorporates hard constraints that guarantee local rigidity. Besides this, the chosen approach also enabled us to achieve clinically feasible runtimes.

### Runtime

Compared with VSA the parametrization used for REG0 achieves almost identical runtimes and is thus in a clinically feasible range. Runtimes for REG1 and REG2 with an average of 28.7 s are assumingly still within an acceptable range, even though computationally heavy anatomical local rigidity constraints are incorporated and the overall deformation plausibility has improved. Especially in comparison with runtimes reported by Kim et al. [18] of about 30–80 min, the runtimes of the presented approach seem more suitable for daily clinical usage.

### Segmentation overlap and displacement regularity

As the presence of a rectal balloon did not impact registration accuracy, VSA and the presented approach seem robust enough to handle these variations. While all tested DIR algorithms showed a significant improvement in accuracy over RIG, all of the three parameterizations of the proposed DIR are well in the range of VSA in terms of DSC. However, in terms of physical plausibility, the presented approach achieves visually superior results. Since the accuracy of VSA is maintained, when choosing between algorithms without rigidity (VSA or REG0) and algorithms with rigidity (REG1 or REG2) the locally rigid approaches are more feasible. Visually this corresponds to the choice between the deformation shown in Fig. 1d and e. Here, while actual benefits in terms of dosimetry or patient outcome are hard to prove, the locally rigid approach gives a result that could represent the real deformation. In contrast, when using the fully deformable approaches, it is apparent that the result can not represent a real deformation. To support this argument, the observed grid foldings represent another class of physically implausible deformations. While here the same argument as before holds that exact effects on patient outcome are hard to show, given the choice between both results, unless tight runtime restrictions apply, the deformation results of REG1 and REG2 should be preferred over VSA. This holds especially for boundary regions of target structures, as grid foldings at organ boundaries, such as those visualized in Fig. 1a, will lead to implausible propagated delineations. It has been shown that correction of propagated structures instead of delineation from scratch can be time saving in a daily clinical workflow [26]. Avoiding such distorted structures may lower the effort a radiation oncologist has to take to manually correct the propagated structures if structure propagation was not sufficient in certain regions. Together with DSC values in the same range as VSA, this could indicate that REG0, REG1 and REG2 will additionally possibly support the manual correction procedure of delineations in the pelvic area. In comparison of REG1 and REG2 there is no clear argument for or against keeping the prostate rigid. Both parameterizations achieve comparable results, both in terms of accuracy and in deformation regularity.

Large bladder volume changes show a limitation of the presented approach. Too large deformations can cause grid foldings, and even though fewer foldings are observed with REG0, REG1 and REG2 than with VSA, they should be avoided completely. For such cases it is beneficial to introduce a specialized constraint that prevents foldings [35]. However, in this work the integration of such constraints was out of scope and is subject to future work.

### Requirements

A requirement of the proposed algorithm are delineations of structures that should be kept rigid on the CT image. However, delineations of important risk structures and CTV areas are routinely performed on a planning CT image and bone structures can be robustly automatically detected in CT images. Therefore in the radiotherapy setting this is barely a limitation. Since it is necessary that the rigid structures are defined on the fixed image, this implies that *I*_CBCT_ is deformed onto *I*_CT_. However, to propagate delineations to *I*_CBCT_ using the resulting deformation as a coordinate transformation, structures can easily be transferred from *I*_CT_ to *I*_CBCT_ without inverting the deformation [20], therefore for most radiotherapy applications this is not a limitation either.

### Parametrization

The examined values for parameters *α*,*τ*,*ϱ* generated a set of values which was used for all evaluations. However, the parameter search showed that slightly different parameters did not yield largely differing DSC values, and therefore a coarse adjustment of the parameters is sufficient. This also supports the assumption that these parameters will remain valid for new, comparable datasets of the same anatomical region and that selected values for *τ* and *ϱ* ensure sufficient filtering of background noise. Additionally, finer deformation resolutions were evaluated, but did not lead to improved results. Here, probably the comparatively low quality CBCT images are limiting registration accuracy [9].

### Comparison to VSA in other studies

To relate the achieved DSC values to other studies, we compare these to results computed with VSA on head/neck and pelvis data. Ramadaan et al. tested VSA in the context of head and neck CT/CT registration [26]. They achieved overall DSC results of 0.82 ± 0.08 and concluded that propagated structures were acceptable for this clinical setting. Thor et al. tested VSA in the context of pelvis CT/CBCT registration [7]. Mean DSC values of 0.80 for prostate and 0.73 for bladder were achieved. In comparison, usage of VSA on our data lead to a mean DSC of 0.79 for prostate and 0.86 for bladder, allowing the conclusion that also REG0, REG1 and REG2 might achieve comparable results on other male pelvis datasets.

### Limitation of gold standard datasets

In comparison to other studies [18], the evaluations performed here were restricted to a single set of gold standard delineations per dataset. Therefore the results might include some observer bias, since, as mentioned above, the inter-observer error is usually rather high regarding DSC values [36]. Because of the extremely difficult and time consuming process of delineating structures (especially prostate) on CBCT images, further redundant delineations by multiple observers were not available. This could be improved in further studies using larger databases and multiple observers.

## Conclusions

The presented DIR framework with local rigidity constraints achieves DSC values comparable to VSA, which suggests that it is maintaining the same accuracy. However, in contrast to VSA, additionally the resulting deformations are physically more plausible, with a largely reduced number of grid foldings and rigid behavior of bones.

Furthermore, the presented algorithm shows that although an advanced deformation model is used, clinically feasible runtimes can be achieved. Therefore, we conclude that the new approach is suitable for radiotherapy applications in the pelvic area, increasing deformation plausibility.

## Additional file

Additional file 1 Mathematical description of the deformable image registration framework. Supplementary appendix which contains additional, detailed mathematical descriptions of the deformable image registration framework. (PDF 126 kb)

## Abbreviations

CBCT: Cone-beam CT
CTV: Clinical target volume
DIR: Deformable image registration
DSC: Dice similarity coefficient
HD: Hausdorff distance
VSA: Varian SmartAdapt
