# Supplementary material for: Deformable image registration for adaptive radiotherapy with guaranteed local rigidity constraints
Source: Radiat Oncol. 2016 Sep 20;11:122. doi: 10.1186/s13014-016-0697-4 (PMC5029058; doi:10.1186/s13014-016-0697-4)
Supplement: Additional file 1 — Mathematical description of the deformable image registration framework. Supplementary appendix which contains additional, detailed mathematical descriptions of the deformable image registration framework. (PDF 126 kb) [file 13014_2016_697_MOESM1_ESM.pdf]

This supplementary appendix complements the Materials & Methods section of the article "Deformable image registration for adaptive radiotherapy with guaranteed local rigidity constraints". It contains additional mathematical descriptions of the presented deformable registration framework.

A

A.1 Registration Framework

Let $\mathcal{R} : \mathbb{R}^3 \to \mathbb{R}$ denote the fixed reference image and $\mathcal{T} : \mathbb{R}^3 \to \mathbb{R}$ the moving template image with compact support in domain $\Omega \subset \mathbb{R}^3$. The goal of image registration is to find a plausible *transformation* $y : \Omega \to \mathbb{R}^3$ that encodes the spatial correspondence between the two images $\mathcal{R}$ and $\mathcal{T}$. In variational approaches, this is modeled by an objective function $\mathcal{J}$ which typically consists of a distance measure $\mathcal{D}$ describing image similarity and a regularizer $\mathcal{S}$ which penalizes implausible deformations [1]. Here, the transformation y is determined by solving the optimization problem

$$\mathcal{J}(y) = \mathcal{D}(\mathcal{R}, \mathcal{T}(y)) + \alpha \mathcal{S}(y) \to \min, \tag{1}$$

where $\alpha > 0$ balances between image similarity and deformation regularity of y and $\mathcal{T}(y) := \mathcal{T} \circ y$ denotes the deformed template image.

Distance Measure Given the CBCT to CT registration problem, the normalized gradient fields distance measure $\mathcal{D}$ is chosen which is suitable for multimodal registration. This distance measure is based on the assumption that in two given images, regardless of modality, object edges and boundaries are always represented by intensity changes, i.e. image gradients. Furthermore it is easy to interpret and can be implemented efficiently and fully parallelized [2, 3]. With $\| \cdot \|_\varepsilon := \sqrt{\langle \cdot, \cdot \rangle + \varepsilon^2}$, we define the NGF distance measure as

$$\mathcal{D}(\mathcal{R}, \mathcal{T}(y)) = \frac{1}{2} \int_\Omega 1 - \left(\frac{\langle \nabla T(y), \nabla R \rangle + \tau \varrho}{\|\nabla T(y)\|_\tau \|\nabla R\|_\varrho} \right)^2 \mathrm{d}x,$$

where $\tau, \varrho \geq 0$ are so-called edge parameters, that allow for image modality specific filtering between noise and relevant image gradients.

Regularizer The second term in the objective function (1) is a regularizer. Here, curvature regularization is used [4]. With the decomposition $y(x) = x + u(x)$, the curvature regularizer is given by

$$\mathcal{S}(y) := \frac{1}{2} \int_\Omega \|\Delta u_x\|^2 + \|\Delta u_y\|^2 + \|\Delta u_z\|^2 \, \mathrm{d}x,$$

where "Δ" denotes the Laplace operator and u_x, u_y, u_z are the components of the displacement in x-, y- and z-direction, respectively.

A.2 Local Rigidity

The framework described in Section A.1 yields a non-linear deformation y that optimizes image similarity based on image gradients and deformation regularity based on second order derivatives. However, these deformations do not incorporate any additional knowledge about special anatomical deformation properties.

Therefore, as proposed in [5] an additional constraint is added to the optimization problem in (1). Given a segmentation of a stiff structure in the planning CT, we define the domain of the segmentation as $\Sigma \subset \Omega$. To model the stiff behavior of this structure, we incorporate a constraint which enforces rigid (i.e. rotational and translational) deformations on this sub-domain. Writing the local rigidity constraint as $\mathcal{C}(y) = 0$ and assuming that it is active on Σ leads to

$$\mathcal{J}(y) = \mathcal{D}(\mathcal{R}, \mathcal{T}(y)) + \alpha\mathcal{S}(y) \rightarrow \min, \qquad \text{s.t.} \quad \mathcal{C}(y)(x) = 0 \quad \text{for all } x \in \Sigma. \tag{2}$$

As there can be several rigid structures acting independently of each other, in general there will be multiple disjoint sub-domains $\Sigma_k, k = 1, \ldots, M$. To restrict structures to rigid deformations only, the constraint term $\mathcal{C}(y)$ is formulated as

$$\begin{aligned} \mathcal{C}(y)(x) &= \mathcal{C}(y, \theta_k, b_k)(x) \\ &= y(x) - (Q(\theta_k)x + b_k) = 0 \\ &\quad \text{for all } x \in \Sigma_k \text{ and } k = 1, \ldots, M, \end{aligned} \tag{3}$$

where Q defines a three-dimensional rotation matrix, depending on the rotation angles $\theta = (\alpha, \beta, \gamma)$ and the components of the translation vector $b = (b_1, b_2, b_3)^\top$.

Constraints elimination Using the formulation in (3), constraints in the optimization problem (2) can be eliminated by substituting the deformation y. Instead of directly optimizing y, all the points that are in some Σ_k are deformed in terms of a rigid deformation and its parameters, yielding

$$y = \begin{pmatrix} y_0 \\ y_1 \\ \vdots \\ y_M \end{pmatrix} = \begin{pmatrix} y_0 \\ Q(\theta_1)x + b_1 \\ \vdots \\ Q(\theta_M)x + b_M \end{pmatrix},$$

where y_0 describes the deformation of all points that are outside of the Σ_k's and thus should deform non-rigidly, while $y_1, \ldots, y_M$ describe rigid deformations of points that are inside $\Sigma_1, \ldots, \Sigma_M$, respectively. Substituting this in the objective function in (2), the optimization problem becomes unconstrained, and can be written as

$$\begin{aligned} \mathcal{J}(y_0, &(\theta_1, b_1), \ldots, (\theta_M, b_M)) = \\ &\mathcal{D}(\mathcal{R}, \mathcal{T}(y_0, (\theta_1, b_1), \ldots, (\theta_M, b_M))) \\ &+ \alpha\mathcal{S}(y_0, (\theta_1, b_1), \ldots, (\theta_M, b_M)) \rightarrow \min . \end{aligned}$$

Here, the optimization is now no longer performed with respect to the deformation, but with respect to a new parameter set $[y_0, (\theta_1, b_1), \ldots, (\theta_M, b_M)]$ where each rigid region Σ_k is controlled by six parameters in θ_k, b_k.

Since each Σ_k is a subset of Ω, the desired rigid areas are defined in the reference image space.

A.3 Discretization and implementation

The optimization problem described in A.1 and A.2 is solved using a *discretize-then-optimize* strategy [1]. The derived continuous model is first discretized on a grid and the obtained function is then optimized using well-established techniques from numerical optimization. To solve the discretized optimization problem, an iterative L-BFGS algorithm [6] is used for minimization. The L-BFGS algorithm is then embedded in a multi-level optimization scheme, where the optimization problem is consecutively solved on coarser to finer grids.

Here, a special strategy is used to prolongate the result obtained on a coarser level to a starting guess on a corresponding fine level. First, the full deformation field is prolongated as it would be using a standard deformable approach. After prolongation a new match of grid points to rigid areas is determined, generating a new set of unconstrained grid points while keeping the same number of rigid parameters $\theta_k, b_k, k = 1, \ldots, M$, since the number of rigid regions does not change. The result from the coarser level is then used as a starting guess for those rigid parameters.

References

[1] Modersitzki, J.: FAIR: Flexible Algorithms for Image Registration. Society for Industrial and Applied Mathematics (SIAM), Philadelphia (2009)

[2] König, L., Rühaak, J.: A fast and accurate parallel algorithm for non-linear image registration using normalized gradient fields. In: Biomedical Imaging (ISBI), IEEE 11th International Symposium On, pp. 580–583 (2014)

[3] König, L., Derksen, A., Hallmann, M., Papenberg, N.: Parallel and memory efficient multimodal image registration for radiotherapy using normalized gradient fields. In: Biomedical Imaging (ISBI), IEEE 12th International Symposium On, pp. 734–738 (2015)

[4] Fischer, B., Modersitzki, J.: Curvature based image registration. J Math Imaging Vis **18**, 81–85 (2003)

[5] Haber, E., Heldmann, S., Modersitzki, J.: A framework for image-based constrained registration with an application to local rigidity. Linear Algebra Appl **431**, 459–470 (2009)

[6] Nocedal, J., Wright, S.J.: Numerical optimization, 2nd edn., pp. 176–180. Springer, New York (1999)
